# A comparative analysis reveals electrogenic properties of PfCRT and pendrin

**DOI:** 10.1016/j.jbc.2025.110630

**Published:** 2025-08-26

**Authors:** Eva Gil-Iturbe, Matthias Quick

**Affiliations:** 1 Department of Psychiatry, Columbia University Irving Medical Center, New York, New York, USA; 2 Department of Physiology & Cellular Biophysics, Columbia University Irving Medical Center, New York, New York, USA; 3 Area Neuroscience - Molecular Therapeutics, New York State Psychiatric Institute, New York, New York, USA

**Keywords:** membrane transport, electrophysiology, sodium-proton exchange, anion transport, drug transport, ion transport, solid-supported membrane, NhaA, PfCRT, pendrin

## Abstract

Ion translocation is an essential process in all living cells. Most traditional approaches studying ion-translocating systems have employed cellular systems replete with native proteins that potentially interfere with the functional assessment of the protein of interest. The reconstitution of purified functional target proteins into proteoliposomes (PLs), artificial membrane systems of defined lipid composition, allows for their characterization without these intricacies. Targeting three distinct proteins, NhaA, pendrin, and the *Plasmodium falciparum* chloroquine resistance transporter (PfCRT), upon their reconstitution into PLs with a combined array of experimental approaches centered around solid-supported membrane electrophysiology, we show the advantage of the PL study system over cell-based approaches to assess protein-specific functional features. Using NhaA, the well-characterized archetype of Na^+^/H^+^ antiporters (exchangers), as a molecular ruler, our studies reveal that pendrin, a clinically relevant anion transporter in the thyroid, ear, kidney, and lungs, catalyzes the electrogenic exchange of its transported anions, opposing a long-standing dogma of the electroneutral activity of pendrin. We also provide direct evidence that PfCRT—a key contributor in multidrug resistance that thwarts efforts to combat malaria—mediates H^+^-coupled drug transport.

Ion flux across biological membranes is an essential process in all living cells. This process is regulated by ion-translocating systems, for example, channels and transporters. Situated in cellular membranes, these systems are essential in regulating central physiological processes, such as cardiac and neuronal activity and the homeostasis of pH, salt, and water, as well as cell proliferation, differentiation, and apoptosis (1). Consequently, several pathological conditions are associated with the dysfunction of ion-translocating systems, such as cystic fibrosis, Bartter's syndrome, or Liddle’s syndrome, among others (2).

Various direct and indirect methods have been employed to study the systems that mediate the specific passage of ions, including electrophysiological techniques, fluorescence spectroscopic methods, radioactive isotope transport assays, and structural biology, as well as molecular dynamics simulations (3, 4, 5, 6). It is generally accepted that conformational changes in ion translocating proteins (referred to as “gating” for channels and “alternating access” for transporters) are the essence of ion translocation across biological membranes (7). Unfortunately, even at high resolution, individual structures of a channel or a transporter, in which the unambiguous attribution of small ions is not always readily attainable, likely capture only a snapshot in a complex mechanistic process. Similarly, molecular dynamics simulations are highly sensitive to initial conditions, system size, and parameter selection, all of which can significantly influence the outcome of mechanistic models. Hence, functional studies are required to elucidate the dynamic elements of ion translocation and to provide guidance for the interpretation of structural and computational models in a functional context.

Major breakthroughs in the functional characterization of ion-translocating systems were achieved with the development of electrophysiological techniques, such as the two-electrode voltage clamp and patch-clamp technique (8), versatile tools that allow the recording of currents associated with ion translocation between compartments (9). The main consideration in these approaches is the minimization of the background electrical noise that interferes with the electrophysiological recordings, notably in patch clamp–associated single-channel current recordings. Ion channels exhibit significantly higher turnover rates compared with transporters, often facilitating the rapid passage of thousands to millions of ions per second. These high flux rates make ion channels particularly well suited for electrophysiological techniques. In stark contrast, transporters operate at much slower rates, typically translocating only a few ions per second, and electrophysiological measurements pose experimental challenges. Transport assays involving radioactive ions offer an alternative approach with robust outcomes. However, radioactive ions are increasingly challenging to obtain from commercial sources, and they pose potential health and environmental risks that demand strict regulatory and administrative compliance.

Furthermore, most traditional approaches employ cellular expression systems that are replete with native proteins that may potentially interfere with the assessment of the protein of interest. To circumvent these potential difficulties, state-of-the-art recombinant protein engineering tools allow for the production and purification of the target protein in fully functional form, thus enabling its incorporation into proteoliposomes (PLs), where functional studies can assess target protein-specific features without these intricacies.

Solid-supported membrane (SSM)–based electrophysiology has played a key role in the functional characterization of numerous transporter systems (6, 10, 11). SSM-based electrophysiology is especially useful when ion translocating proteins are incorporated in artificial membrane systems and cannot be functionally characterized in cellular expression systems because of the noise generated by native cellular proteins or when these systems are situated in intracellular membranes. SSM employs a synthetic solid-supported lipid bilayer membrane, onto which the membrane-incorporated protein of interest is adsorbed. A rapid exchange of the assay medium from the “nonactivating” (NA) condition to the “activating” (A) condition triggers ion transport events in the test system that cause a change of the membrane potential. In contrast to “conventional” electrophysiological measurements that display charge transfer within or across the membrane electric field as the function of time, SSM recordings report the change of the membrane potential associated with net ion flux across the membrane (3).

Here, we report the ion flux activity of three distinct proteins using SSM electrophysiology in conjunction with radiotracer- and fluorescence-based uptake studies: NhaA, pendrin, and the *Plasmodium falciparum* chloroquine (CQ) resistance transporter (PfCRT). These transporters are involved in crucial physiological and pathophysiological processes, such as ion homeostasis and pH regulation and multidrug resistance. NhaA is the primary regulator of Na^+^ and H^+^ homeostasis in *Escherichia coli* and is considered the archetype of Na^+^/H^+^ antiporters (exchangers) (12). Pendrin is an anion transporter widely expressed in the thyroid gland, inner ear, kidney, and lungs, where it plays important roles in maintaining ion balance and fluid homeostasis (13, 14, 15). The PfCRT is located in the parasite's digestive vacuole (DV) membrane. It was first recognized as the primary factor responsible for CQ resistance (16), and it is now recognized as a key contributor in multidrug resistance against 4-aminoquinoline (4-AQ) compounds to efficiently combat malaria (17).

## Results and discussion

To assess mechanistic details of ion transport by NhaA, pendrin, and PfCRT reconstituted into PLs, we performed SSM-based electrophysiological measurements, radiolabeled substrate uptake, and fluorescence-based measurements of intraliposomal pH changes. For the SSM measurements, we used the SURFE^2^R N1 platform (Nanion Technologies, GmbH). Here, PLs containing NhaA, pendrin, or PfCRT were absorbed on lipid membrane–coated gold sensors. A rapid solution exchange introduces the substrate, triggering transport events. As ions flow into the PLs, capacitive coupling between the sensor and the PLs is recorded (18, 19, 20). Radiotracer uptake studies were performed using a rapid filtration-based method. Here, the amount of radioactivity accumulated in PLs that are retained on the filter is detected with scintillation counting, whereas free radioactivity from the assay medium is passed through the filter in the filtrate (6). For the fluorescence-based activity measurements, PLs were preloaded with Oregon Green 514, a pH-sensitive dye with a low p*K*a that reports changes of the intraliposomal pH. Alterations of the internal pH are reflected in the dye's fluorescence intensity that can be used to quantify the intraliposomal H^+^ accumulation that is directly associated with transport activity. Performing these different methods under controlled experimental conditions in parallel serves as internal methodological validation and guidance for the assessment of ion transport by each of the targeted proteins.

### Na^+^/H^+^ antiport by NhaA

Taking advantage of NhaA as a well-established model system that was previously characterized in intact bacterial cells, in bacterial membrane vesicles, and in purified and reconstituted form using fluorescence-, radiotracer-, and SSM-based approaches (3, 12, 21), we used NhaA as a molecular ruler for our SSM electrophysiological measurements. In addition to the wealth of functional information of NhaA, the high-resolution structural analysis revealed its assembly with the highly conserved residues D163 and D164 located in the proposed Na^+^-binding site (22, 23, 24), providing guidance for functional analyses in a structural context. A key mechanistic finding relevant for the SSM studies on NhaA is the fact that NhaA mediates the electrogenic exchange of one Na^+^ for two H^+^ across the membrane in opposite directions (*i.e.*, antiport) (25).

**Figure 1:**
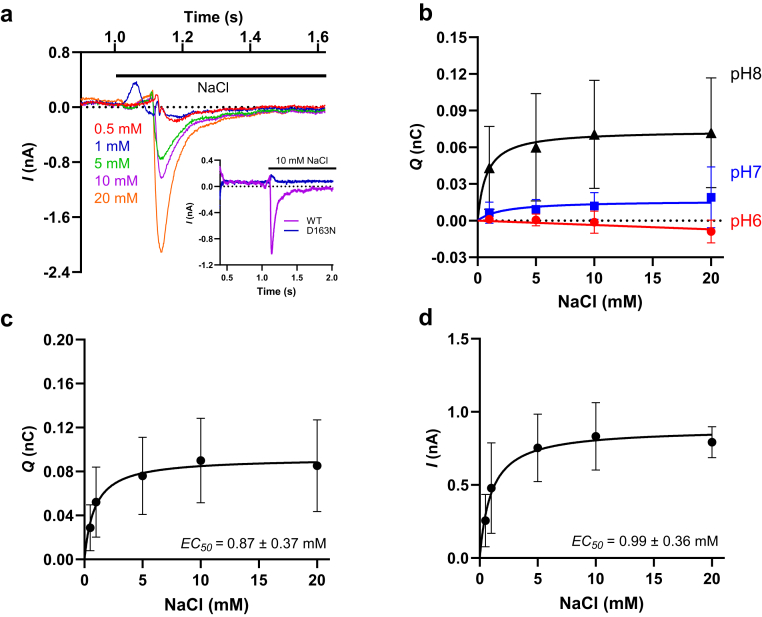
**Measurements in proteoliposomes containing NhaA**. *A*, representative recording of an SSM electrophysiological measurement of WT NhaA performed in the SURFE^2^R N1 system. *Inset*, recorded currents of WT NhaA and the no functional mutant, D163N, after 10 mM NaCl was added at time 1 s. The *black bar* shows the addition of different concentrations of NaCl as indicated in the legend. *B*, transport kinetics of NaCl using proteoliposomes containing NhaA at different pHs. *C*, transport kinetics of NaCl using the area under the currents obtained from SSM electrophysiological measurement of WT NhaA (control liposomes used for signal correction) at pH 8.0. Fitting the data to a nonlinear regression model yielded an apparent affinity for Na^+^ of 0.87 ± 0.37 mM. *D*, transport kinetics of NaCl using the maximal currents obtained from SSM electrophysiological measurement of WT NhaA (control liposomes used for signal correction) at pH 8.0. Fitting the data to a nonlinear regression model yielded an apparent affinity for Na^+^ of 0.99 ± 0.36 mM. All data are mean ± SD (n = 5–7 independent biological replicates), and the currents of three individual sensors were integrated over time to determine the transfer of charges (Coulombs) associated with protein-specific transport by determining the area under the curve using GraphPad Prism 10. SSM, solid-supported membrane.

Figure 1*A* shows a typical current recording using the SURFE^2^R N1 system. When 10 mM NaCl was added to the sensor containing PL with NhaA-WT, a downward current was generated. The downward deflection of the current in this assay format is indicative of the net move of a positive charge out of the PLs or the net move of a negative charge into the PLs, and it indicates here that NhaA transports one Na^+^ inward in exchange with the outward transport of two H^+^ from inside the PLs (26). In contrast, the inactive D163N mutant showed no signal, in agreement with previous reports indicating a lack of Na^+^ binding (12) or antiport activity (23).

NhaA operates with a remarkably rapid catalytic turnover rate (>1000 s^-1^) and is highly sensitive to pH changes, a property it shares with eukaryotic Na^+^/H^+^ antiporters (25). NhaA has been reported to be active if ≥pH 8.0 and inactive if ≤pH 6.5 (12, 27). To confirm its pH sensitivity, we conducted SSM experiments at pH values of 6.0, 7.0, and 8.0 (Fig. 1*B*). Consistent with previous findings, NhaA exhibited maximal transport activity at pH 8.0, and no signal was detected at pH 6.0, consistent with previous SSM studies (27) and other studies employing cells and bacterial membrane vesicles and PL (27, 28, 29, 30).

We further investigated the apparent *K*_*m*_ for Na^+^ by varying NaCl concentrations in the external assay medium. Plotting Na^+^-induced charge transfer from the SSM-based experiments as a function of the external Na^+^ concentration yielded an apparent *K*_*m*_ of 0.87 ± 0.37 mM at pH 8.0 (Fig. 1*C*). This value is similar to the apparent *K*_*m*_ determined in previous studies using NhaA-containing intact bacterial cells, bacterial cell membrane preparations, and PLs (25, 30, 31, 32, 33). It aligns with the 0.5 mM cytoplasmic Na^+^ concentration that NhaA maintains in the bacteria (12, 34). Moreover, a recent study using scintillation proximity assay similarly reported apparent Na^+^-binding affinities around 0.5 mM (12). We note, however, that the apparent *K*_*m*_ of 0.87 mM at pH 8.0 in our measurements is about 10-fold lower than that reported in previous reports using the SSM technology (>10 mM, (11, 27)). In contrast to these publications (3, 11, 27, 35), where the maximum current (nA) was used to determine the apparent *K*_*m*_, we plotted the total charge (in nC) to calculate the apparent *K*_*m*_. To rule out that the readout of the measurements (nC *versus* nA) affects the results, we also plotted the measured nA in our experiments as a function of the Na^+^ concentration (Fig. 1*D*). The results of this calculation (using nA) yielded a similar apparent *K*_*m*_ when compared with that determined when nC was used for the readout in our assays (apparent *K*_*m*_ of 0.99 ± 0.36 mM at pH 8.0).

### Electrogenic anion transport by pendrin

Pendrin, a member of the SLC26 family of anion transporters, is an anion exchanger that mediates the translocation of chloride (Cl^-^), iodide (I^-^), formate (CHO_2_^-^), bicarbonate (HCO_3_^-^), and hydroxide (OH^-^) across epithelial cells, most notably the thyroid, kidney, and inner ear (13, 14, 15). Mutations in pendrin are linked to several genetic disorders, such as Pendred's syndrome and enlarged vestibular aqueduct syndrome, characterized by congenital hearing loss and the development of a goiter because of impaired thyroid hormone synthesis (15, 36). In addition, overexpression of pendrin has been observed in conditions such as chronic obstructive pulmonary disease, cystic fibrosis, and some respiratory infections (37).

**Figure 2:**
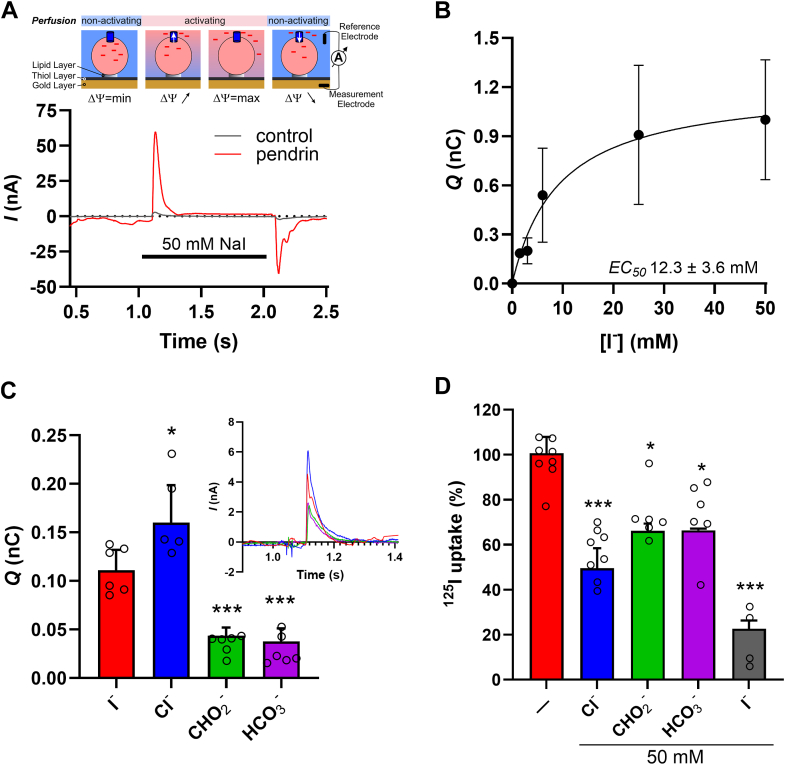
**Pendrin-mediated anion transport.***A*, representative SSM recordings of pendrin-specific on-signal and control proteoliposome (PL) (absence of protein) current traces in response to the addition of 50 mM NaI (*black bar*) to assay buffer at time 1 s. *Inset*, schematic representation of the concept of the SSM measurements (10). *B*, transport kinetics of Nal using the currents obtained from SSM electrophysiological measurement of PLs containing pendrin. Integration of the on-signal currents (control liposomes used for signal correction) over time reveals the pendrin-specific charge transfer. Fitting the data (mean ± SD, n = 6 independent biological replicates) to a nonlinear regression model yielded an apparent affinity for iodide anions of 12.3 ± 3.6 mM. *C*, integration of the on-signal currents over time reveals the pendrin-specific charge transfer in response to Na^+^-salts. *Inset*, a representative SSM recording of pendrin PL. Data are mean ± SD (n = 6 independent biological replicates) of the area under the peak. *D*, 1-min uptake of 10 μM Na^125^I was measured in pendrin-PLs in the absence (−) or presence of 50 mM Cl^-^, CHO_2_^-^, HCO_3_^-^, or l^-^. Data (mean ± SD, n = 6 independent biological replicates) uptake was corrected for unspecific uptake in control liposomes and normalized with regard to the 1-min uptake in the presence of l^-^. ∗*p* < 0.05, ∗∗*p* < 0.01. Statistical tests and exact *p* values are provided in Table S1. SSM, solid-supported membrane.

Figure 2*A* shows a typical SSM current when 50 mM NaI was added in the activating condition to pendrin-containing PLs or control liposomes that lack protein. The addition of NaI elicited a large pendrin-specific upward deflection of the current recordings, reflective of the change in the membrane potential in response to the efflux of negative charges from the pendrin-containing PLs. Since no anions known to be transported by pendrin were incorporated in the internal buffer of the pendrin-containing PLs, this suggests that pendrin can mediate the electrogenic exchange of I^-^ (influx) and OH^-^ (efflux) in the PLs. This result is consistent with previous reports of pendrin exchanging Cl^−^ and OH^−^ in cultured human embryonic kidney 293 (HEK293) cells (38) and in the perfused proximal tubule (39, 40). However, the fact that the I^-^ flux–induced export of OH^-^ yielded such a large current signal indicates that the pendrin-mediated anion exchange mechanism cannot be electroneutral as previously predicted (41). Similar to NhaA, where the influx of 1 Na^+^ is associated with the efflux of 2 H^+^, our data point to pendrin as an electrogenic anion exchanger with a stoichiometry larger than unity for the pendrin-mediated fluxes of anions in different directions.

We further investigated the effect of varying I^-^ concentrations of the SSM-based recordings under the same experimental settings. Plotting the I^-^-induced charge transfer in the SSM-based experiments as a function of the external I^-^ concentration yielded an apparent EC_50_ for I^-^ of 12.3 ± 3.6 mM (Fig. 2*B*), a value comparable with previous determinations of the affinity of I^-^ uptake by pendrin expressed in *Xenopus* oocytes (42, 43) and in PLs (37). To test the anion specificity of pendrin, we equimolarly replaced 50 mM I^-^ in the assay buffer with anionic substrates. Our measurements revealed the following order of specificity: Cl^-^ ≥ I^-^ > CHO_2_^-^ = HCO_3_^-^ (Fig. 2*C*). This specificity pattern was also observed in uptake studies in PLs (Fig. 2*D*), where we measured 1-min uptakes of ^125^I^-^ in the presence of 50 mM of the same anionic substrates. In other studies that measured the exchange of H[^14^C]O_3_ with 100 mM anionic substrates inside the PLs, a slightly different specificity was observed, with Cl^-^ > CHO_2_^-^ > I^-^ (37). A previous study performed in *Xenopus* oocytes expressing pendrin indicated that pendrin functions as a Cl^−^/HCO_3_^-^, Cl^−^/I^−^, and I^−^/HCO_3_^-^ exchanger, preferring I^-^ as a substrate (42).

**Figure 3:**
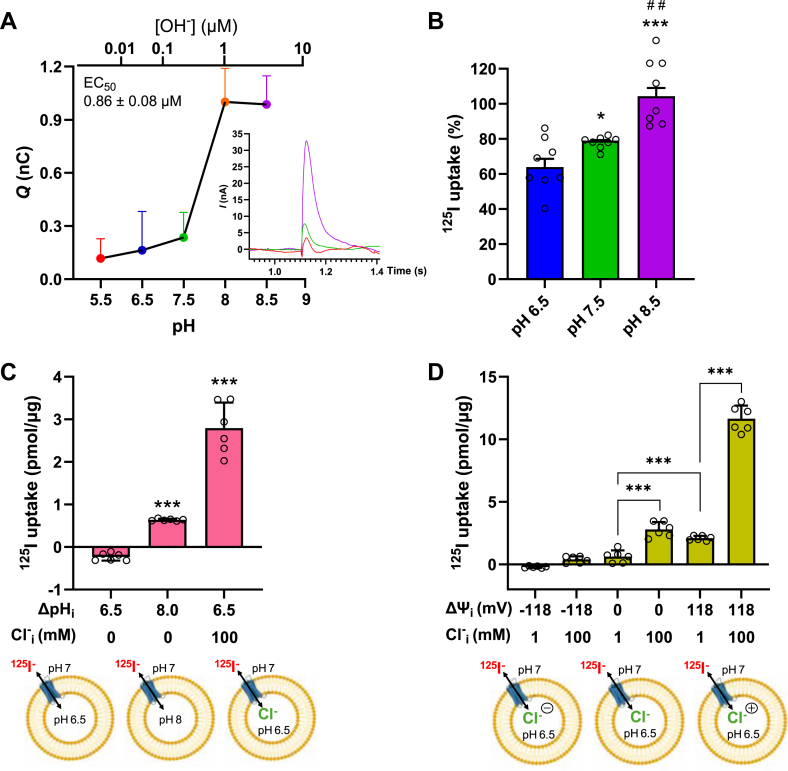
**Pendrin-mediated ^125^I^-^ transport is electrogenic and pH dependent.***A*, normalized charge transfer elicited by the addition of 25 mM I^-^ to external assay buffer (pH 7.5) in response to varying the internal pH in the proteoliposomes (PLs). *Inset*, representative pendrin-specific on-signal current traces in response to the addition of 25 mM NaI to assay buffer composed of 200 mM Tris–Gly-Gly, at pH 5.5, 7.5, or 8.5. Fitting the data (mean ± SD, n = 4 independent biological replicates) to a nonlinear regression model yielded an apparent affinity for hydroxide anions (OH^-^) of 0.86 ± 0.08 μM (or a pOH of 6.07 or a pH of 7.93). *B*, transport of 10 μM ^125^I^-^ was measured in pendrin-containing PLs for 1-min periods in assay buffer composed of 200 mM Tris–Gly-Gly, at pH 6.5, 7.5, or 8.5. Data (mean ± SD, n = 6 independent biological replicates) are normalized with regard to pH 8.5. *C*, transport of 10 μM ^125^I^-^ was measured in pendrin-containing PLs for 1-min periods in assay buffer composed of 200 mM Tris–Gly-Gly, pH 7. The PLs were preloaded with 200 mM Tris–Gly-Gly, pH 6.5, pH 8.0, or pH 6.5, and 100 mM NaCl (equimolar replacement with Tris–Gly-Gly) as indicated in the scheme. *D*, 1-min uptakes were measured with pendrin-containing PLs preloaded with 100 mM Tris–Gly-Gly, pH 6.5 and 100 mM KCl (100 mM Cl^-^_i_), 100 mM Tris–Gly-Gly, pH 6.5 and 1 mM NaCl/99 mM KCl (100 mM Cl^-^_i_), or 100 mM Tris–Gly-Gly, pH 6.5 and 99 mM K-gluconate/1 mM KCl (1 mM Cl^-^_i_) and diluted into assay buffer composed of 100 mM Tris–Gly-Gly, pH 7.0/100 mM K-gluconate or 199 mM Tris–Gly-Gly, pH 7.0/1 mM K-gluconate in the presence or absence of the K^+^ ionophore valinomycin (1 μM) plus 10 μM ^125^I^-^. The scheme illustrates the experimental conditions. Data are means ± SD (n = 6 independent biological replicates).∗*p* < 0.05, ∗∗∗*p* < 0.001 *versus* pH 6.5 l^-^, #*p* < 0.05 *versus* pH 7.5. Statistical tests and exact *p* values are provided in Table S1.

Furthermore, we tested the effect of the internal pH in the pendrin-containing PLs on electrical signal while maintaining a constant external pH of 7.5 (Fig. 3*A*). The largest currents were observed when the internal pH was ≥8.0. Plotting the currents elicited upon the addition of external I^-^ as a function of the internal OH^-^ concentration revealed a strict dependence of the activity of pendrin on the pH. Fitting the data to a nonlinear regression model yielded an apparent affinity for OH^-^ of 0.86 ± 0.08 μM (corresponding to a pH of 7.93 or a pOH of 6.07). This trend was also observed in ^125^I^-^ uptake studies in PLs at different pH values (Fig. 3*B*). Similar findings were reported in a HEK293 cellular heterologous expression system, where the apparent Cl^−^ affinity worsened with a lower internal pH (44). Physiologically, the relatively high affinity at high pH values would enable modulation of HCO_3_^-^/OH^-^ secretion and regulate the alkalosis in tissues, such as the kidney, inner ear, or lung. In addition, another Cl^−^/HCO_3_^-^ anion exchanger, like AE2, appears to be inhibited slightly by acidic pH and more significantly activated by high pH (45).

Figure 3*C* shows that ^125^I^-^ uptake (influx) required either a pH or Cl^-^ gradient across the PL membrane. The uptake increased three times when the PLs were preloaded with a more basic buffer (pH 8.0) than the external assay medium (pH 7.0) or 13-fold when we preloaded the PL with 100 mM Cl^-^ at pH 6.5, whereas the assay medium was virtually free of Cl^-^. Other studies have similarly demonstrated increased H[^14^C]O_3_ and ^125^I^-^ uptakes when 150 mM Cl^−^ were included in the PL, suggesting thermodynamic coupling of Cl^−^ and HCO_3_^-^ exchange (37).

By using potassium-filled PLs, a membrane potential (△Ψ) could be created by adding the K^+^-specific ionophore, valinomycin. The valinomycin-generated K^+^-diffusion potential–dependent ΔΨ, where, according to the Nernst equation, a 10-fold concentration difference results in a membrane potential of 59 mV (46, 47). Our data reveal that the I^-^ uptake activity increased fourfold when both an outward-directed Cl^-^ gradient and an inside-positive K^+^-diffusion potential–generated △Ψ of 118 mV were imposed across the PL membrane (Fig. 3*D*). Substantial and comparable I^-^ uptake activity was observed when either the pendrin-containing PLs were preloaded with 100 mM Cl^-^ and △Ψ = 0 mV or when an inside-positive △Ψ of 118 mV was generated in PLs that were preloaded with 1 mM Cl^-^ (Fig. 3*D*). An inside-negative △Ψ precluded significant I^-^ transport regardless of the Cl^-^ concentration inside of the PLs or when △Ψ was 0 mV and the inside Cl^-^ concentration was 1 mM. Consistent with previous reports, our transport data in the PL system suggest that pendrin mediates the exchange (antiport) of I^-^ and Cl^-^. Supporting the findings of our SSM measurements, and in contrast to the long-standing notion that pendrin-mediated anion exchange is electroneutral (38, 42), our uptake data give rise to the hypothesis that the pendrin-mediated transport reaction is in fact electrogenic. Our recent structural determination of pendrin reveals the existence and functional importance of two anion-binding sites in pendrin (37). This structural observation, in combination with our new findings pointing to the electrogenic transport mechanism of pendrin, does warrant further studies to delineate the role of the individual anion sites in the overall transport mechanism for this protein implicated in several clinical conditions and targeted for therapeutic application.

### PfCRT-mediated H^+^ transport

Genetic variations in the *pfcrt* gene, coding for PfCRT that is situated in the intraerythrocytic parasite’s DV membrane, are directly responsible for *P. falciparum* resistance to 4-AQ antimalarials, such as CQ and piperaquine (PPQ) (48). In addition to its proposed function to act as a conduit for the transport of various molecules such as peptides and iron from the degradation of the host’s heme, the export of 4-AQ compounds from the DV manifests the role of PfCRT as a promising drug target (49, 50). According to the weak base hypothesis, CQ and PPQ, as weak bases, can passively permeate cellular membranes and accumulate in the acidic DV of the intraerythrocytic parasite as protonated species (CQ^2+^ and PPQ^4+^), where they bind to toxic Fe^3+^–heme and inhibit heme incorporation into chemically inert hemozoin (51). Mutations in the *pfcrt* gene are directly associated with the parasite's ability to evade antimalarial drug action. For example, the 7G8 isoform that dominates in South America and the Western Pacific area includes five amino acid mutations that allow it to transport CQ in a membrane potential– and pH-dependent manner; however, it does not transport PPQ (48). Functional studies on the PfCRT 7G8 variant that also harbors the additional F145I and C350R mutations revealed a decreased PPQ susceptibility in Asia and South America, respectively, thus emphasizing the ability of additional mutations to modulate 4-AQ transport activity (52).

**Figure 4:**
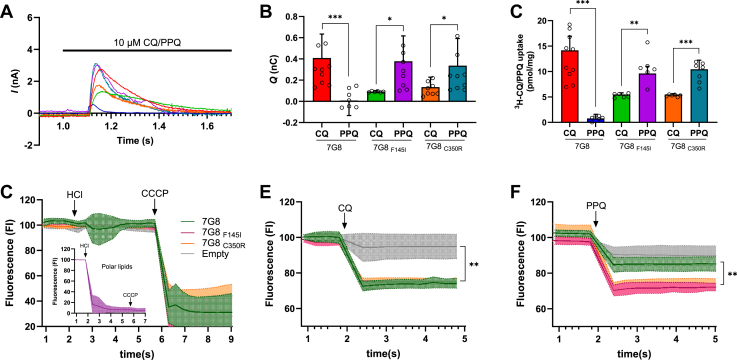
**Measurements in proteoliposomes (PLs) containing PfCRT**. *A*, representative SSM recordings of PLs containing indicated PfCRT variants upon the addition of 10 μM of CQ (7G8 in *red*, 7G8_F145I_ in *green*, and 7G8_C350R_ in *orange*) or PPQ (7G8 in *dark blue*, 7G8_F145I_ in *purple*, and 7G8_C350R_ in *blue*). CQ and PPQ were added to the assay buffer as indicated by the *arrow*. *B*, ttransport of 10 μM CQ or PPQ using PLs containing PfCRT with a K^+^ diffusion potential–driven membrane potential and inwardly directed pH gradient (pH_in_ = 7.5; pH_out_ = 5.5). Data are mean ± SD (n = 6–9 independent biological replicates) of the area under the peak. Currents were integrated over time to determine the transfer of charges (Coulombs) associated with protein-specific transport by determining the area under the curve using GraphPad Prism 10. *C*, transport of 100 nM ^3^H-CQ and ^3^H-PPQ was measured for 30 s using PLs containing PfCRT with a K^+^ diffusion potential–driven membrane potential and inwardly directed pH gradient (pH_in_ = 7.5; pH_out_ = 5.5). Data are mean ± SD (n = 6 independent biological replicates). Uptake by PfCRT-containing PLs was corrected for unspecific uptake in control liposomes and data. *D*, proton flux measurements. PfCRT PLs were loaded with 10 μM Oregon *Green* 514 carboxylic acid, and the fluorescence was monitored. In the presence of 0.03 M HCl, the addition of the ionophore CCCP, that disrupts the pH gradient, rapidly reduces the fluorescence to the baseline level. *Inset*, polar lipid liposomes under the same conditions. *E* and *F*, 100 μM CQ or PPQ. For consistency with previous measurements (48), a K^+^ diffusion potential–driven membrane potential was generated with valinomycin, and an inwardly directed pH gradient (pH_in_ = 7.5; pH_out_ = 5.5) was present in *A*–*F*. Data are mean ± SD (n = 5–6 independent biological replicates). ∗*p* < 0.05, ∗∗*p* < 0.01, and ∗∗∗*p* < 0.001. Statistical tests and exact *p* values are provided in Table S1. CQ, chloroquine; PfCRT, *Plasmodium falciparum* chloroquine resistance transporter; PPQ, piperaquine; SSM, solid-supported membrane.

To test whether PfCRT-mediated drug transport is associated with charge movement, we performed SSM-based measurements with PLs containing PfCRT variants 7G8, 7G8_F145I,_ or 7G8_C350R_. Figure 4*A* shows typical SURFE^2^R N1-based current recordings when 10 μM of CQ or PPQ was added to the assays with the different PfCRT isoforms. Consistent with the current recordings in NhaA and pendrin, the upward deflection of the current traces upon the addition of substrate (CQ or PPQ) to PfCRT-containing PLs in an assay buffer that was shown to support ^3^H-CQ or ^3^H-PPQ transport in the respective isoforms (48), reflects the change of the membrane potential in response to the accumulation of positive charges within the PLs. We conclude that this PfCRT-specific charge transfer is reflective of the electrogenic influx of positively charged H^+^ in the PLs. In PfCRT 7G8, the addition of CQ generated an upward peak, whereas PPQ did not, as expected for this PPQ-sensitive and CQ-resistant isoform. On the other hand, in the other two tested mutants (7G8_F145I_ and 7G8_C350R_), PPQ elicited currents that were three times higher than those observed with CQ (Fig. 4*B*). The same pattern was also observed in PL-based uptake studies using ^3^H-CQ or ^3^H-PPQ (Fig. 4*C*). We previously described the same pattern of radiolabeled CQ/PPQ uptake in PLs and intracellular accumulation ratio for CQ after 1 h in *P. falciparum* isoforms (7G8, 7G8_F145I,_ and 7G8_C350R_) (48). Other studies also showed that in the CQ-resistant PfCRT isoforms, the F145I and C350R mutations conferred PPQ resistance and substantially reduced growth rates (53, 54, 55).

We observed in the SSM measurements that the area under the current, reflective of the charge movement, is comparable between CQ and PPQ. However, protonated PPQ (PPQ^4+^) has double the charge than CQ (CQ^2+^), suggesting that the change transfer we detect in the SSM measurements cannot reflect the transport of the charged drug itself but positive charges that move much faster than the accumulation of CQ or PPQ. Note that ^3^H-CQ or ^3^H-PPQ transport activity peaks within a time range of about 30 s to 1 min in the PL and *P. falciparum* test systems (48), and we conclude that the observed charge movement in the SSM recordings displays the translocation of freely moving H^+^. Several studies have suggested that the transport of drugs *via* PfCRT is associated with H^+^ and is dependent on the membrane potential (49, 56, 57). To further evaluate the movement of H^+^ in the PfCRT-containing PLs, we preloaded the PL and control liposomes with Oregon Green 514 carboxylic acid, a pH-sensitive dye, and measured the change in fluorescence. The addition of HCl reduced the pH of the solution and led to a rapid fluorescence reduction in control liposomes made of *E. coli* polar lipids (Fig. 4*D*, *inset*), indicating that under these extreme conditions, protons can permeate the membrane composed of *E. coli* polar lipids. This finding aligns with observations by Tsai and Miller (58), which revealed that low concentrations of HCl appear to be the carrier of H^+^ into the liposomes, consistent with previous observations that the lipid permeability of HCl is ∼10^9^ times higher than that of H^+^ (59, 60). In contrast, in our PfCRT PL preparations (or control liposomes) consisting of *E. coli* total lipids and cholesteryl hemisuccinate (CHS) (at a 97:3 w/w ratio), the pH gradient across the liposome membrane is maintained upon the addition of HCl (Fig. 4*D*). However, when the protonophore carbonyl cyanide *m*-chlorophenyl hydrazone is added to the assay medium, H^+^ freely move across the membrane and rapidly reduces the fluorescence in the PLs and liposomes made of *E. coli* total lipids and CHS. Consequently, these data show that our PL preparations can maintain a transmembrane H^+^ gradient for several minutes, which is enough time for the transport experiments contemplated in the study. Moreover, we use the same conditions to investigate the movement of H^+^ after the addition of CQ and PPQ. Figure 4*E* shows a 30% reduction of fluorescence after adding 100 μM CQ in all PfCRT-containing PLs, except in control liposomes. In agreement with the SSM and radiolabeled uptake experiments (Fig. 4, *B* and *C*) and parasite PPQ susceptibility studies (52, 55), the largest fluorescence reduction upon the addition of 100 μM PPQ was observed for the PfCRT variants 7G8_F145I_ and 7G8_C350R_ (Fig. 4*F*).

Here, we present a comparative analysis of ion-translocating systems using several experimental approaches on NhaA, pendrin, and PfCRT to correlate flux measurements with SSM-based electrophysiological recordings. Collectively, these studies underscore the value of using electrophysiological, radiolabeled substrate transport, and fluorescence-based approaches to unravel the functional specifics of ion-transporting systems. Taken together, our data, while validating established NhaA functional characteristics, identified that pendrin performs the electrogenic exchange of its respective anionic substrates. This finding stands in contrast to the long-standing proposal of electroneutral anion exchange by this SLC26 family member. However, in light of our recent structural discovery of two anionic sites in pendrin (37), it is interesting to speculate as to how the potential interaction of the two anion-binding sites during the transport process is correlated to the electrogenic exchange mechanisms and how known inhibitors of pendrin interfere with this mechanism.

Similarly, our combined array of SSM electrophysiology, radiotracer-based uptake measurements, and fluorescence-based recordings provide strong support for the H^+^-coupled transport of 4-AQ drugs, for example, H^+^/drug symport. By taking advantage of purified recombinant PfCRT variants reconstituted into PLs, a system devoid of potentially interfering proteins found in native cellular systems, we were able to decipher the PfCRT-specific mechanistic elements associated with drug transport.

## Experimental procedures

### Protein purification

#### NhaA

His-tagged NhaA was overexpressed from the pAXH355 plasmid in *E. coli* TA16 as previously described (12). Briefly, cells were cultured in minimal medium A supplemented with 0.5% (w/v) glycerol, 0.01% (w/v) MgSO_4_·7 H_2_O, and 2.5 μg/ml thiamine and 100 μg/ml ampicillin, and protein expression was induced at mid1log phase with isopropyl β-d-1-thiogalactopyranoside. Following a 2-h induction, cells were harvested, and high-pressure membrane vesicles were prepared. NhaA was purified *via* Ni^2+^-affinity chromatography and eluted in a buffer containing 10% glycerol, 300 mM imidazole, 25 mM citric acid, 100 mM choline chloride, 5 mM MgCl_2_, and 0.015% *n*-dodecyl-β-d-maltopyranoside (DDM). The eluate was supplemented with sucrose (10%), dialyzed overnight at 4 °C in acidic buffer (25 mM potassium citrate [pH 4.0], 10% sucrose, 100 mM choline chloride, 25 mM citric acid, 5 mM MgCl_2_, and 0.015% DDM), and stored at −80 °C until further use.

### PfCRT and pendrin

The PfCRT and *Sus scrofa* pendrin, each containing a C-terminal His-tag, were expressed and purified as previously described (37, 48). PfCRT and pendrin were cloned into the pEG BacMam and pFastBac vectors, respectively, and expressed using the Bac-to-Bac system (Thermo Fisher Scientific). Recombinant P1 baculovirus was used to infect Sf9 insect cells for initial amplification. For PfCRT expression, P4 virus was subsequently used to infect HEK293S GnTi^-^ mammalian cells, whereas pendrin was expressed in Sf9 cells.

Membrane fractions were isolated from HEK293S GnTi^-^ cells for PfCRT and from Sf9 cells for pendrin. Membranes were solubilized at 4 °C for 2 h with gentle agitation using either 1% (w/v) DDM and 0.1% (w/v) CHS for PfCRT or 1.5% (w/v) lauryl maltose neopentyl glycol (LMNG) and 0.01% CHS for pendrin. Insoluble material was removed by ultracentrifugation, and the supernatant was incubated with Ni^2+^–NTA affinity resin (Qiagen) at 4 °C. Following extensive washing, PfCRT was eluted in a buffer containing 200 mM imidazole, 0.05% DDM, and 0.005% CHS, whereas pendrin was eluted using buffer supplemented with 300 mM imidazole, 0.1% LMNG, and 0.01% CHS. Protein samples were shock frozen in liquid nitrogen and stored at −80 ^o^C until use.

### Preparation of PLs

*E. coli* total or polar lipids (Avanti Polar Lipids, Inc.) and CHS (Sigma–Aldrich) were used to prepare PLs. Functional preparations of purified *E. coli* NhaA (in 25 mM potassium citrate, pH 4.0, 10% sucrose, 100 mM choline chloride, 25 mM citric acid, 5 mM MgCl_2_, 0.015% [w/v] DDM (12), PfCRT [in 20 mM Hepes], pH 7.5, 200 mM NaCl, 200 mM imidazole, 0.05% [w/v] DDM with 0.005% [w/v] CHS (48), and *S. scrofa* pendrin [in 20 mM Hepes, 150 mM NaCl, 2 mM β-mercaptoethanol, 1  mM PMSF, and 0.01% [w/v] LMNG (37)) were used for reconstitution at a protein-to-lipid ratio of 1:150 (w/w). Unless indicated otherwise, *E. coli* total lipids were used for the preparation of NhaA-containing PLs (or control liposomes lacking NhaA), and *E. coli* total lipids and CHS (97:3 w/w) were used for PLs containing pendrin or PfCRT (or for the preparation of control liposomes). The lipids were resuspended in a buffer containing *n*-octyl-β-d-glucopyranoside to form presolubilized lipid micelles, and *n*-octyl-β-d-glucopyranoside was removed by dialysis to generate preformed liposomes (61). For the reconstitution of the target proteins, preformed liposomes were partially solubilized by the addition of Triton X-100, and then the detergent was removed by the stepwise addition of Bio-Beads SM-2 (Bio-Rad). PLs were frozen in liquid nitrogen and stored at −80 ^o^C until use for functional assays. The lumen of the NhaA PLs was composed of 15 mM Tris–Cl, pH 7.5/6.5, 150 mM NH_4_Cl, and 1 mM DTT. The pendrin-containing PLs were prepared in 100 mM Tris–Gly-Gly, pH 6.4, and the PfCRT-containing PLs were prepared in 100 mM potassium phosphate (KP_i_), pH 7.5, and 2 mM β-mercaptoethanol. To maintain their stability, the PLs were aliquoted, rapidly frozen in liquid nitrogen, and stored at −80 °C.

## SSM-based electrophysiology

Electrophysiology measurements were conducted using the SURFE^2^R N1 system (Nanion Technologies GmbH), a platform designed to detect electrogenic transport events through capacitive current recordings. This technique relies on the detection of transient charge displacements that occur upon rapid substrate addition to PLs adsorbed to an SSM sensor. For a comprehensive protocol and technical background, see the studies by Bazzone and Barthmes (10) and Pommereau *et al.* (62). The *inset* in Figure 2, a illustrates the principle of the SSM measurements. Prior to measurements, the sensors were processed following the manufacturer’s guidelines and subjected to quality control by measuring their conductance and capacitance (10). In brief, the sensors’ gold surface was coated with 1-octadecanethiol (Sigma–Aldrich), rinsed and dried, and 1,2-diphytanoyl-*sn*-glycero-3-phosphocholine (Avanti Polar Lipids, Inc) was added to form the SSM. Instantly, the sensor was filled with NA buffer along with protein-containing PLs. Prior to use, the PLs underwent extrusion and sonication as described (48). Then, sensors were centrifuged to promote adsorption of the PLs to the SSM.

For measurements, the sensors were placed into the Faraday cage of the SURFE^2^R N1, and a single-solution exchange protocol was applied. In each cycle, the NA and activating (A) buffers were exchanged in the sequence NA-A-NA, with a 1-s incubation in the activating buffer (with the substrates indicated in each figure). Individual measurements were performed at least six times with at least three different sensors. Peak currents were corrected by subtracting the corresponding currents recorded with control liposomes (lacking protein).

Peak currents reflect the rapid displacement of charge because of transporter-mediated ion movement across the membrane of the PLs. At the same time, the sensor charges as well. Unless otherwise noted, currents were integrated over time to determine the transfer of charges (Q, Coulombs) associated with protein-specific signals by determining the area under the curve using GraphPad Prism 10 (GraphPad Software, Inc). These values were used to quantify transporter activity and determine apparent affinities for various substrates.

The composition of the NA and A buffers varied depending on the transporter under investigation. For NhaA, 10 mM Tris–Cl, pH 8.6, 150 mM choline chloride, and 25 mM MgSO_4_ was used as NA buffer. The NA buffer for pendrin was 200 mM Tris–Gly-Gly, pH 5.5 to 8.5. For PfCRT, 100 mM Tris–Mes, pH 5.5 with 80 nM valinomycin was used, as previously described (48). In the A buffers, the substrates were added (equimolar replacement with choline chloride, Tris–Gly-Gly, or Tris–Mes, respectively) as indicated in the figure legends.

### Transport measurements

The uptake of ^125^I^-^ (0.1/0.5 Ci/mol), ^3^H-CQ, and ^3^H-PPQ (1 Ci/mmol) was measured with a rapid filtration assay using 0.45 μM nitrocellulose filters (Millipore) as described (37). PLs (30 ng protein per assay) were diluted in 50 μl of uptake buffer in the presence or absence of compounds as indicated in the figure legends. Reactions were quenched by the rapid addition of ice-cold 100 mM KP_i_, pH 6.0, and 100 mM LiCl after 30 s before filtration. The radioactivity retained on the filters was determined with scintillation counting (Hidex SL300 scintillation counter) using the dried filters. Known amounts of radioactivity were used to convert decays per minute to mol.

### Proton flux measurements

PLs composed of *E. coli* polar lipids containing PfCRT or control liposomes lacking PfCRT were used to assess proton flux based on internal pH changes. PLs were loaded with the pH-sensitive fluorescent dye Oregon Green 514 carboxylic acid to monitor internal pH changes. PLs were loaded with 10 μM Oregon Green 514 carboxylic acid (Invitrogen), a pH-sensitive fluorescent dye, by subjecting them to repeated freeze–thaw cycles followed by brief sonication to promote dye encapsulation. Excess extraliposomal dye was removed by gel filtration using Sephadex G-50 columns equilibrated with the internal buffer. The purified (proteo)liposomes were then diluted 10-fold in external assay buffer prior to measurement.

Fluorescence measurements were performed in black, flat-bottom 96-well plates using a PHERAstar FS plate reader (BMG Labtech), with excitation at 489 nm and emission at 524 nm. Changes in fluorescence intensity reflect changes in the internal pH of the liposomes, with a drop in fluorescence intensity reflecting intraliposomal acidification. To initiate proton flux, the external pH was rapidly lowered by adding 0.03 M HCl to reduce the solution pH to ∼2.2 to 3.0, generating a transmembrane pH gradient. Proton influx into the liposome lumen leads to quenching of Oregon Green fluorescence, allowing real-time monitoring of proton transport or proton leakage across the membrane. The ionophore cyanide *m*-chlorophenyl hydrazone (1 μg/ml) was added at the end of the time course to collapse the pH gradient, confirming that fluorescence changes were due to proton gradients and membrane permeability rather than photobleaching or dye leakage. All measurements were performed at least five times using independent liposome preparations. Fluorescence traces were normalized to the initial value (set to 100) to allow comparison between replicates.

### Statistical analysis

Statistical analyses were performed using GraphPad Prism (version 10). Data distribution was assessed using the Shapiro–Wilk test to determine normality. For comparisons between two groups, either a two-tailed unpaired *t* test (for normally distributed data) or the Mann–Whitney *U* test (for non-normally distributed data) was applied. For kinetics analyses, nonlinear regression was used to fit the Michaelis–Menten model. All statistical tests used and corresponding *p* values are provided in Table S1.

## Data availability

The data analyzed in the current study are available from the corresponding author upon reasonable request.

## Supporting information

This article contains supporting information.

## Conflict of interest

The authors declare that they have no conflicts of interest with the contents of this article.

## Supporting information

Supplemenatary Information

## References

[1] Remigante A., Gavazzo P., Morabito R., Dossena S. (2022). Editorial: ion transporters and channels in cellular pathophysiology. Front. Cell Dev. Biol..

[2] Farley R.A., Sperelakis N. (2012). Cell Physiology Source Book.

[3] Dong F., Lojko P., Bazzone A., Bernhard F., Borodina I. (2024). Transporter function characterization *via* continuous-exchange cell-free synthesis and solid supported membrane-based electrophysiology. Bioelectrochemistry.

[4] Howard R.J., Carnevale V., Delemotte L., Hellmich U.A., Rothberg B.S. (2018). Permeating disciplines: overcoming barriers between molecular simulations and classical structure-function approaches in biological ion transport. Biochim. Biophys. Acta Biomembr..

[5] Cui G., Cottrill K.A., McCarty N.A. (2021). Electrophysiological approaches for the study of ion channel function. Methods Mol. Biol..

[6] Dvorak V., Wiedmer T., Ingles-Prieto A., Altermatt P., Batoulis H., Barenz F. (2021). An overview of cell-based assay platforms for the solute carrier family of transporters. Front. Pharmacol..

[7] Reuss L. (2008). Seldin and Giebisch’s The Kidney.

[8] Cahalan M., Neher E. (1992). Patch clamp techniques: an overview. Methods Enzymol..

[9] Pun R.Y.K., Lecar H. (1995). Cell Physiology Source Book.

[10] Bazzone A., Barthmes M. (2020). Functional characterization of SLC transporters using solid supported membranes. Methods Mol. Biol..

[11] Ganea C., Fendler K. (2009). Bacterial transporters: charge translocation and mechanism. Biochim. Biophys. Acta.

[12] Quick M., Dwivedi M., Padan E. (2021). Insight into the direct interaction of Na(+) with NhaA and mechanistic implications. Sci. Rep..

[13] Bizhanova A., Kopp P. (2010). Genetics and phenomics of pendred syndrome. Mol. Cell Endocrinol.

[14] Alper S.L., Sharma A.K. (2013). The SLC26 gene family of anion transporters and channels. Mol. Aspects Med..

[15] Seidler U., Nikolovska K. (2019). Slc26 family of anion transporters in the gastrointestinal tract: expression, function, regulation, and role in disease. Compr. Physiol..

[16] Blasco B., Leroy D., Fidock D.A. (2017). Antimalarial drug resistance: linking Plasmodium falciparum parasite biology to the clinic. Nat. Med..

[17] Ferreira L.T., Cassiano G.C., Alvarez L.C.S., Okombo J., Calit J., Fontinha D. (2024). A novel 4-aminoquinoline chemotype with multistage antimalarial activity and lack of cross-resistance with PfCRT and PfMDR1 mutants. PLoS Pathog..

[18] Bazzone A., Costa W.S., Braner M., Calinescu O., Hatahet L., Fendler K. (2013). Introduction to solid supported membrane based electrophysiology. J. Vis. Exp..

[19] Schulz P., Garcia-Celma J.J., Fendler K. (2008). SSM-based electrophysiology. Methods.

[20] Bazzone A., Barthmes M., Fendler K. (2017). SSM-Based electrophysiology for transporter research. Methods Enzymol..

[21] Patino-Ruiz M., Dwivedi M., Calinescu O., Karabel M., Padan E., Fendler K. (2019). Replacement of Lys-300 with a glutamine in the NhaA Na^+^/H^+^ antiporter of *Escherichia coli* yields a functional electrogenic transporter. J. Biol. Chem..

[22] Hunte C., Screpanti E., Venturi M., Rimon A., Padan E., Michel H. (2005). Structure of a Na+/H+ antiporter and insights into mechanism of action and regulation by pH. Nature.

[23] Inoue H., Noumi T., Tsuchiya T., Kanazawa H. (1995). Essential aspartic acid residues, Asp-133, Asp-163 and Asp-164, in the transmembrane helices of a Na+/H+ antiporter (NhaA) from Escherichia coli. FEBS Lett..

[24] Winkelmann I., Uzdavinys P., Kenney I.M., Brock J., Meier P.F., Wagner L.M. (2022). Crystal structure of the Na(+)/H(+) antiporter NhaA at active pH reveals the mechanistic basis for pH sensing. Nat. Commun..

[25] Padan E. (2014). Functional and structural dynamics of NhaA, a prototype for Na(+) and H(+) antiporters, which are responsible for Na(+) and H(+) homeostasis in cells. Biochim. Biophys. Acta.

[26] Taglicht D., Padan E., Schuldiner S. (1993). Proton-sodium stoichiometry of NhaA, an electrogenic antiporter from Escherichia coli. J. Biol. Chem..

[27] Mager T., Rimon A., Padan E., Fendler K. (2011). Transport mechanism and pH regulation of the Na+/H+ antiporter NhaA from Escherichia coli: an electrophysiological study. J. Biol. Chem..

[28] Tzubery T., Rimon A., Padan E. (2008). Structure-based functional study reveals multiple roles of transmembrane segment IX and loop VIII-IX in NhaA Na+/H+ antiporter of Escherichia coli at physiological pH. J. Biol. Chem..

[29] Venturi M., Rimon A., Gerchman Y., Hunte C., Padan E., Michel H. (2000). The monoclonal antibody 1F6 identifies a pH-dependent conformational change in the hydrophilic NH(2) terminus of NhaA Na(+)/H(+) antiporter of Escherichia coli. J. Biol. Chem..

[30] Taglicht D., Padan E., Schuldiner S. (1991). Overproduction and purification of a functional Na+/H+ antiporter coded by nhaA (ant) from Escherichia coli. J. Biol. Chem..

[31] Tzubery T., Rimon A., Padan E. (2004). Mutation E252C increases drastically the Km value for Na+ and causes an alkaline shift of the pH dependence of NhaA Na+/H+ antiporter of Escherichia coli. J. Biol. Chem..

[32] Padan E., Schuldiner S. (1993). Na+/H+ antiporters, molecular devices that couple the Na+ and H+ circulation in cells. J. Bioenerg. Biomembr..

[33] Goldberg E.B., Arbel T., Chen J., Karpel R., Mackie G.A., Schuldiner S. (1987). Characterization of a Na^+^/H^+^ antiporter gene of *Escherichia coli*. Proc. Natl. Acad. Sci. U. S. A..

[34] Harel-Bronstein M., Dibrov P., Olami Y., Pinner E., Schuldiner S., Padan E. (1995). MH1, a second-site revertant of an Escherichia coli mutant lacking Na+/H+ antiporters (delta nhaA delta nhaB), regains Na+ resistance and a capacity to excrete Na+ in a delta microH(+)-independent fashion. J. Biol. Chem..

[35] Zuber D., Krause R., Venturi M., Padan E., Bamberg E., Fendler K. (2005). Kinetics of charge translocation in the passive downhill uptake mode of the Na+/H+ antiporter NhaA of Escherichia coli. Biochim. Biophys. Acta.

[36] Dossena S., Nofziger C., Tamma G., Bernardinelli E., Vanoni S., Nowak C. (2011). Molecular and functional characterization of human pendrin and its allelic variants. Cell Physiol. Biochem..

[37] Wang L., Hoang A., Gil-Iturbe E., Laganowsky A., Quick M., Zhou M. (2024). Mechanism of anion exchange and small-molecule inhibition of pendrin. Nat. Commun..

[38] Soleimani M., Greeley T., Petrovic S., Wang Z., Amlal H., Kopp P. (2001). Pendrin: an apical Cl-/OH-/HCO3- exchanger in the kidney cortex. Am. J. Physiol. Ren. Physiol..

[39] Kurtz I., Nagami G., Yanagawa N., Li L., Emmons C., Lee I. (1994). Mechanism of apical and basolateral Na(+)-independent Cl-/base exchange in the rabbit superficial proximal straight tubule. J. Clin. Invest..

[40] Sheu J.N., Quigley R., Baum M. (1995). Heterogeneity of chloride/base exchange in rabbit superficial and juxtamedullary proximal convoluted tubules. Am. J. Physiol..

[41] Liu Q., Zhang X., Huang H., Chen Y., Wang F., Hao A. (2023). Asymmetric pendrin homodimer reveals its molecular mechanism as anion exchanger. Nat. Commun..

[42] Shcheynikov N., Yang D., Wang Y., Zeng W., Karniski L.P., So I. (2008). The Slc26a4 transporter functions as an electroneutral Cl-/I-/HCO3- exchanger: role of Slc26a4 and Slc26a6 in I- and HCO3- secretion and in regulation of CFTR in the parotid duct. J. Physiol..

[43] Reimold F.R., Heneghan J.F., Stewart A.K., Zelikovic I., Vandorpe D.H., Shmukler B.E. (2011). Pendrin function and regulation in Xenopus oocytes. Cell Physiol. Biochem..

[44] Azroyan A., Laghmani K., Crambert G., Mordasini D., Doucet A., Edwards A. (2011). Regulation of pendrin by pH: dependence on glycosylation. Biochem. J..

[45] Sterling D., Casey J.R. (1999). Transport activity of AE3 chloride/bicarbonate anion-exchange proteins and their regulation by intracellular pH. Biochem. J..

[46] Szabo G., Eisenman G., Laprade R., Ciani S.M., Krasne S. (1973). Experimentally observed effects of carriers on the electrical properties of bilayer membranes--equilibrium domain. With a contribution on the molecular basis of ion selectivity. Membranes.

[47] Jung H., Tebbe S., Schmid R., Jung K. (1998). Unidirectional reconstitution and characterization of purified Na+/proline transporter of Escherichia coli. Biochemistry.

[48] Kim J., Tan Y.Z., Wicht K.J., Erramilli S.K., Dhingra S.K., Okombo J. (2019). Structure and drug resistance of the Plasmodium falciparum transporter PfCRT. Nature.

[49] Bakouh N., Bellanca S., Nyboer B., Moliner Cubel S., Karim Z., Sanchez C.P. (2017). Iron is a substrate of the Plasmodium falciparum chloroquine resistance transporter PfCRT in Xenopus oocytes. J. Biol. Chem..

[50] Shafik S.H., Cobbold S.A., Barkat K., Richards S.N., Lancaster N.S., Llinas M. (2020). The natural function of the malaria parasite's chloroquine resistance transporter. Nat. Commun..

[51] Ecker A., Lehane A.M., Clain J., Fidock D.A. (2012). PfCRT and its role in antimalarial drug resistance. Trends Parasitol..

[52] Ross L.S., Dhingra S.K., Mok S., Yeo T., Wicht K.J., Kumpornsin K. (2018). Emerging Southeast Asian PfCRT mutations confer Plasmodium falciparum resistance to the first-line antimalarial piperaquine. Nat. Commun..

[53] Hagenah L.M., Dhingra S.K., Small-Saunders J.L., Qahash T., Willems A., Schindler K.A. (2024). Additional PfCRT mutations driven by selective pressure for improved fitness can result in the loss of piperaquine resistance and altered Plasmodium falciparum physiology. mBio.

[54] Wicht K.J., Small-Saunders J.L., Hagenah L.M., Mok S., Fidock D.A. (2022). Mutant PfCRT can mediate piperaquine resistance in African Plasmodium falciparum with reduced fitness and increased susceptibility to other antimalarials. J. Infect. Dis..

[55] Pelleau S., Moss E.L., Dhingra S.K., Volney B., Casteras J., Gabryszewski S.J. (2015). Adaptive evolution of malaria parasites in French Guiana: reversal of chloroquine resistance by acquisition of a mutation in pfcrt. Proc. Natl. Acad. Sci. U. S. A..

[56] Sanchez C.P., Stein W.D., Lanzer M. (2007). Is PfCRT a channel or a carrier? Two competing models explaining chloroquine resistance in Plasmodium falciparum. Trends Parasitol..

[57] Lehane A.M., Hayward R., Saliba K.J., Kirk K. (2008). A verapamil-sensitive chloroquine-associated H+ leak from the digestive vacuole in chloroquine-resistant malaria parasites. J. Cell Sci..

[58] Tsai M.F., Miller C. (2013). Substrate selectivity in arginine-dependent acid resistance in enteric bacteria. Proc. Natl. Acad. Sci. U. S. A..

[59] Nozaki Y., Tanford C. (1981). Proton and hydroxide ion permeability of phospholipid vesicles. Proc. Natl. Acad. Sci. U. S. A..

[60] Finkelstein A. (1976). Water and nonelectrolyte permeability of lipid bilayer membranes. J. Gen. Physiol..

[61] Rigaud J.L., Pitard B., Levy D. (1995). Reconstitution of membrane proteins into liposomes: application to energy-transducing membrane proteins. Biochim. Biophys. Acta.

[62] Pommereau A., Licher T., Barenz F. (2023). Solid-supported membrane (SSM)-Based electrophysiology assays using surface electrogenic event reader Technology (SURFE(2)R) in early drug discovery. Curr. Protoc..

