# Supplementary material for: A comparative analysis reveals electrogenic properties of PfCRT and pendrin
Source: J Biol Chem. 2025 Aug 26;301(10):110630. doi: 10.1016/j.jbc.2025.110630 (PMC12494547; doi:10.1016/j.jbc.2025.110630)
Supplement: Supplemenatary Information [file mmc1.docx]

**Supporting information**

Table 1: Statistical tests and exact *P* values were obtained using GraphPad Prism (version 10).  *P* < 0.05 indicated statistical significance.
